# Supplementary material for: Identification of photoperiod-induced specific miRNAs in the adrenal glands of Sunite sheep (Ovis aries)
Source: Front Vet Sci. 2022 Jul 22;9:888207. doi: 10.3389/fvets.2022.888207 (PMC9354845; doi:10.3389/fvets.2022.888207)
Supplement: Supplementary file 1 [file Data_Sheet_1.ZIP › Supplementary file/miRDeep2 file.zip!/miRDeep2 file/Novel_Structure/SP42A1/Novel_325.pdf]

Provisional ID	:	NC_019484.2_26676
Score total	:	2.5
Score for star read(s)	:	-1.3
Score for read counts	:	0
Score for mfe	:	2.2
Score for randfold	:	1.6
Score for cons. seed	:	
Total read count	:	30
Mature read count	:	28
Loop read count	:	0
Star read count	:	2

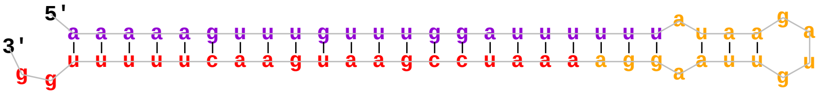

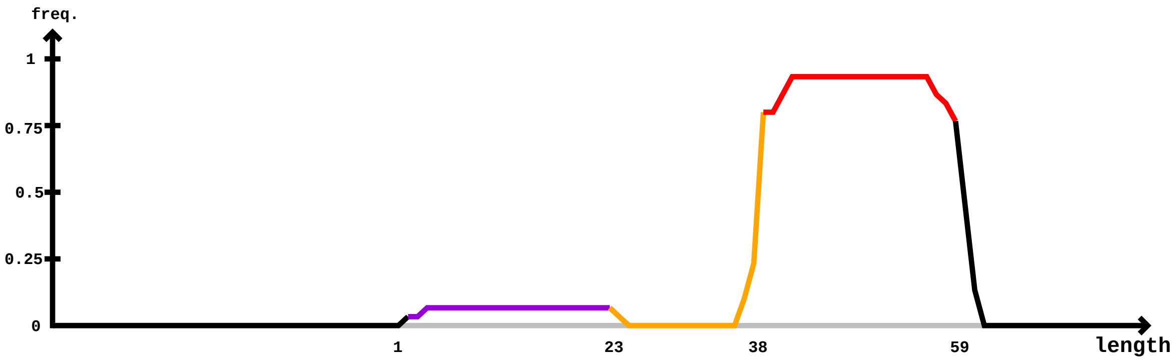


```
                                   Star                               Mature
5'- uacaugugcgaauuguuacuaauauugggcuggccaaaaaguuuguuuggauuuuuuauaagauguuaaggaaaauccgaaugaacuuuuuggccugcccaauacaucugcc   -3'    obs
    uacaugugcgaauuguuacuaauauugggcuggccaaaaaguuuguuuggauuuuuuauaagauguuaaggaaaauccgaaugaacuuuuuggccugcccaauacaucugcc          exp
    ......................(((((((.(((((((((((((((((((((((((((.(((....))).)))))))))))))))))))))))))).))))))))........    reads     mm      sample
    ...................................aaaaaguuuguuuggGuuuuuu.......................................................    1         1       seq
    .....................................aaaguuuguuuggGuuuuuu.......................................................    1         1       seq
    ......................................................................gaaaauccgaaugaacuuuu......................    1         0       seq
    ......................................................................gaaaauccgaaugaacuuuuu.....................    1         0       seq
    ......................................................................gaaaauccgaaugaacuuuuug....................    1         0       seq
    .......................................................................aaaauccgaaugaacuuuu......................    1         0       seq
    .......................................................................aaaauccgaaugaacuuuuug....................    1         0       seq
    .......................................................................aaaauccgaaugaacuuuuugg...................    2         0       seq
    ........................................................................aaaCccgaaugaacuuuuugg...................    17        1       seq
    ..........................................................................auccgaaugaacuuuuuggUc.................    1         1       seq
    ..........................................................................aCccgaaugaacuuuuuggcc.................    1         1       seq
    ...........................................................................uccgaaugaacuuuuuggUc.................    2         1       seq
```
